# An Increase in Mitochondrial DNA Promotes Nuclear DNA Replication in Yeast

**DOI:** 10.1371/journal.pgen.1000047

**Published:** 2008-04-25

**Authors:** Heidi M. Blank, Chonghua Li, John E. Mueller, Lydia M. Bogomolnaya, Mary Bryk, Michael Polymenis

**Affiliations:** Department of Biochemistry and Biophysics, Texas A&M University, College Station, Texas, United States of America; National Institute of Diabetes and Digestive and Kidney Diseases, United States of America

## Abstract

Coordination between cellular metabolism and DNA replication determines when cells initiate division. It has been assumed that metabolism only plays a permissive role in cell division. While blocking metabolism arrests cell division, it is not known whether an up-regulation of metabolic reactions accelerates cell cycle transitions. Here, we show that increasing the amount of mitochondrial DNA accelerates overall cell proliferation and promotes nuclear DNA replication, in a nutrient-dependent manner. The Sir2p NAD^+^-dependent de-acetylase antagonizes this mitochondrial role. We found that cells with increased mitochondrial DNA have reduced Sir2p levels bound at origins of DNA replication in the nucleus, accompanied with increased levels of K9, K14-acetylated histone H3 at those origins. Our results demonstrate an active role of mitochondrial processes in the control of cell division. They also suggest that cellular metabolism may impact on chromatin modifications to regulate the activity of origins of DNA replication.

## Author Summary

How cells determine when to divide is critical for understanding biological processes where cell proliferation is manifest. Because cells need to accumulate precursors prior to duplication, cellular metabolism is expected to impact cell division. Mitochondrial processes are central to the control of overall cell metabolism. Yet, the mechanisms that link mitochondrial processes with nuclear DNA replication remain largely unknown. We found that budding yeast cells moderately over-expressing Abf2p, a mtDNA maintenance protein, accelerate nuclear DNA replication. These cells with more mitochondrial DNA proliferate and increase in size more rapidly than their wild type counterparts. The results suggest that cells over-expressing Abf2p have up-regulated metabolic functions, which in turn enable these cells to accelerate initiation of cell division. We also examined the role of Sir2p, an NAD^+^-dependent de-acetylase, which negatively controls DNA replication. We found that the level of Sir2p bound at origins of DNA replication is inversely related to the amount of mtDNA in the cell. In summary, our findings challenge the notion that metabolic processes are required for cell division by simply operating at constitutive background levels. Instead, our work suggests that mitochondrial transactions can actively promote DNA replication and cell division.

## Introduction

Without cellular metabolism there is no cell division [1], but the key question is whether metabolism only allows for division to happen, or can it actively promote cell cycle progression. To determine if metabolism can actively promote cell division it is important to identify gain-of-function mutations in metabolic pathways that also accelerate cell proliferation. Such mutations have not been described in the yeast *Saccharomyces cerevisiae*. It is known, however, that yeast populations evolved in continuous chemostat cultures can proliferate faster than the parent population, and they have higher levels of tricarboxylic acid cycle (TCA) enzymes and mitochondrial (mt) DNA [2]. The mitochondrial genome is transmitted as a “nucleoid” DNA/protein complex. The number of mtDNA molecules per nucleoid varies, but there are usually more genome equivalents than nucleoids [3],[4]. Abf2p is a conserved mtDNA maintenance protein [5],[6], which directly binds to, bends and compacts mtDNA [7],[8]. Moderate over-expression of Abf2p by 2–3 fold elevates the amount of mtDNA by 50–150% [9]. The consequences of an increase in mtDNA in cell proliferation have not been explored.

Sir2p is an evolutionarily conserved NAD^+^-dependent de-acetylase [10],[11]. Loss of Sir2p leads to loss of transcriptional silencing, genome instability and a decrease in replicative life span. In yeast, silent chromatin is formed at three regions: the rDNA, the *HML* and *HMR* mating type loci, and telomeres [12]. Sir2p is required for silencing at all of these regions, and it is the only Sir protein required for silencing at the rDNA [13]–[15]. Sir2p also appears to negatively impact on rDNA replication, because in *sir2Δ* cells twice as many origins are activated within the rDNA array [16]. The inhibitory effects of Sir2p on DNA replication extend beyond rDNA. Loss of Sir2p suppresses replication defects of mutants that cannot assemble a pre-replicative complex of proteins (pre-RC) at origins of DNA replication in the G1 phase of the cell cycle [17]. These results may be linked to a general positive role of histone acetylation for origin activity [18]. Indeed, loss of the Rpd3p de-acetylase globally accelerated DNA replication, and targeted acetylation of a late origin advanced its activation [19], demonstrating a clear causal role of histone acetylation and activation of DNA replication. However, whether such chromatin modifications may serve as a link between cellular metabolism and initiation of DNA replication is not known.

In this report we show that an increase in mtDNA in cells over-expressing Abf2p, actively promotes initiation of cell division. Furthermore, we identify physical changes, such as Sir2p binding and histone acetylation, at an origin of DNA replication that result from an increase in mtDNA.

## Results/Discussion

### Abf2p and Cell Proliferation in Chemostats

**Figure 1:**
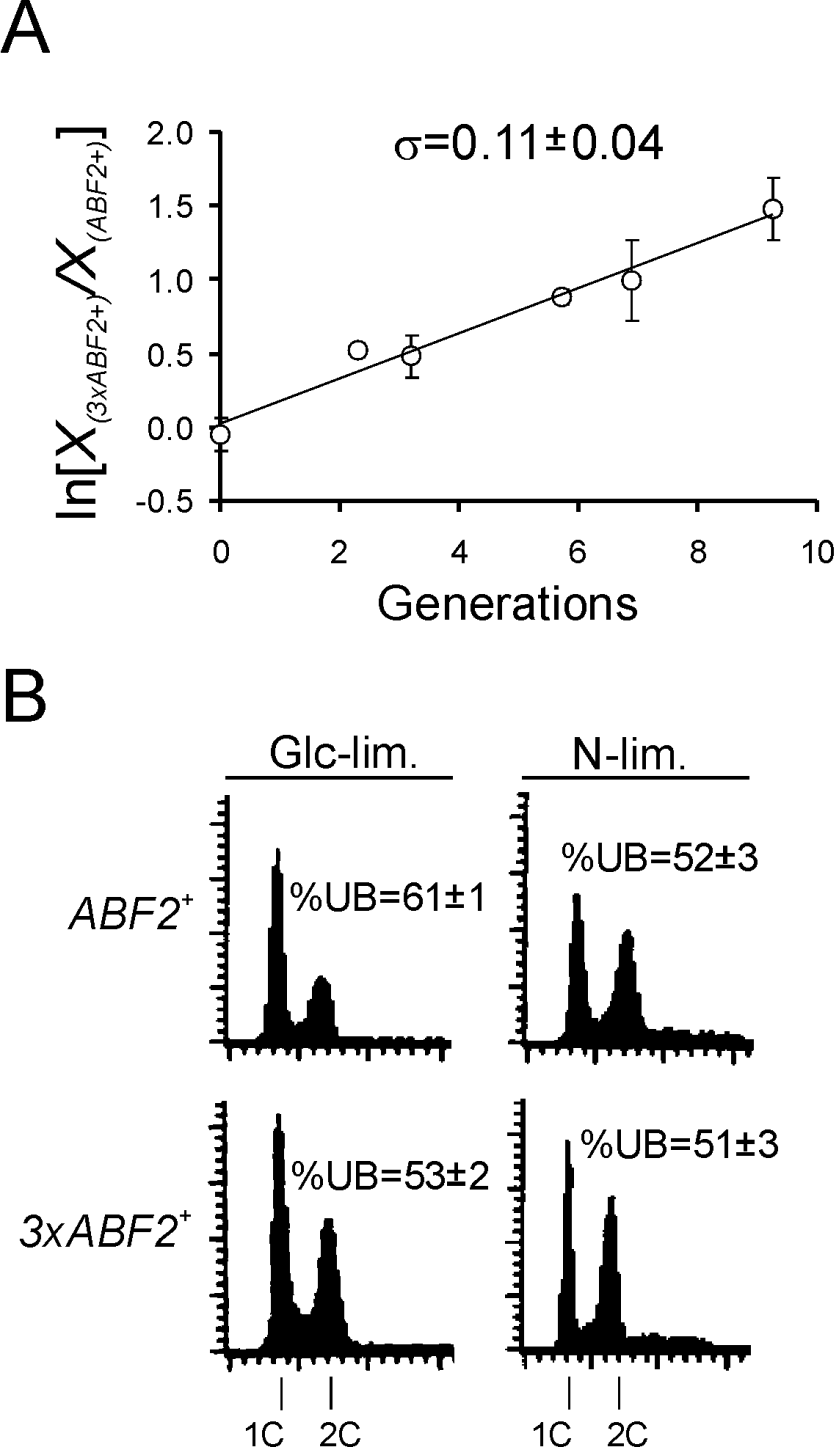
Cells moderately over-expressing *ABF2* proliferate faster (*A*), and have altered cell cycle progression (*B*) in chemostat cultures. In *A*, a chemostat competition experiment done during glucose limitation at dilution rate *D* = 0.1 h^−1^ is shown. The specific growth differential (σ) between the two competing strains [41] is the average (±s.d.) of three independent competition experiments. In each experiment, and for each sampling point, the average (±s.d.) of at least three measurements was calculated. X*_3xABF2_^+^*(t) and X*_ABF2_^+^*(t) represent the relative proportion of the two strains at time t, in generations. In *B*, cell cycle progression of the indicated strains and nutrient limitations was evaluated by flow cytometry. Cell numbers are plotted on the y-axis and fluorescence on the x-axis. The DNA content of cells in G1 and G2/M is indicated as 1C and 2C, respectively. The percentage of unbudded cells (%UB) correlates with G1 cells. The values shown are the average (±s.d.) of at least three independent experiments.

**Figure 2:**
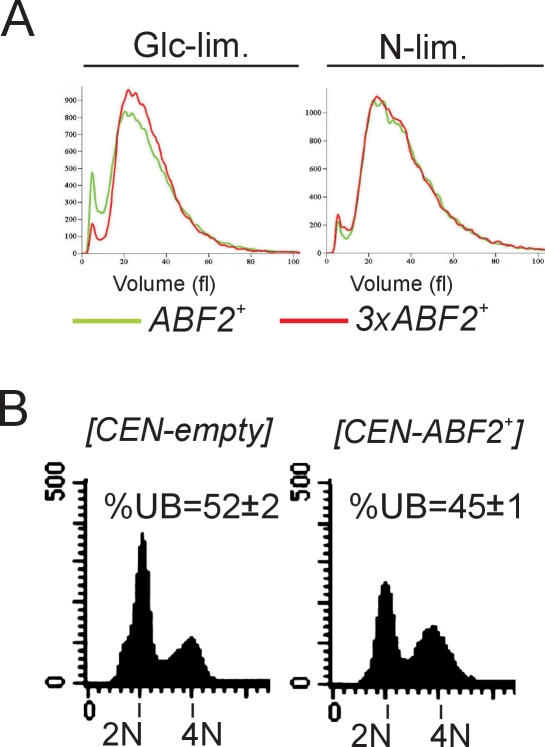
*3xABF2^+^* cells do not have altered cell size in chemostat cultures. *A*, The cell size of the indicated cell populations was measured from the same chemostat experiments described in Figure 1B, using a channelyzer. Cell numbers are plotted on the y-axis and the x-axis indicates size (in fl). *B*, Moderate over-expression of *ABF2* from a low-copy CEN plasmid promotes cell cycle progression. The DNA content of the indicated strains is shown.

We hypothesized that increasing the amount of mtDNA may mimic the situation of “evolved” yeast populations, which can proliferate faster than the parent population [2], allowing us to examine effects on cell division. We evaluated a strain (*3xABF2^+^*), which carries two additional copies of *ABF2*, because in this strain the amount of mtDNA is increased [9]. The *3xABF2^+^* strain proliferated faster than the wild type strain in glucose-limiting (0.08% glucose) conditions (Figure 1A), mimicking “evolved” strains [2]. We next examined cell cycle progression in defined chemostat cultures under glucose (Glc) or nitrogen (N) limitation at 0.2 h^−1^ dilution rate, *D*, comparing *ABF2^+^* to *3xABF2^+^* cells (Figure 1B). Under Glc-limitation, the fraction of *3xABF2^+^* cells in G1 was reduced (Figure 1B, cells in G1, 53% *3xABF2^+^* compared to 61% *ABF2^+^*). In contrast, under N-limitation, extra copies of *ABF2* did not affect the DNA content distribution in anabolically-restricted cells (Figure 1B, cells in G1, 52% *3xABF2^+^* compared to 51% *ABF2^+^*). These data suggest a connection between mitochondrial function and cell cycle progression that is evident under glucose limitation in cells over-expressing *ABF2*. Interestingly, *3xABF2^+^* cells are the same size as wild type cells (Figure 2A), possibly explaining why *ABF2* mutations were not identified previously in size-based mutant screens for cell cycle regulators.

To determine whether the effects observed in cell cycle progression are due to *ABF2* and not due to an unknown secondary mutation, we repeated the experiment shown in Figure 1B using a different genetic background (BY4743). We used a low-copy centromeric plasmid carrying *ABF2* (*CEN-ABF2^+^*) to achieve modest over-expression of Abf2p similar to *3xABF2* ^+^ cells [9],[20]. Under glucose limitation, a lower fraction of *CEN-ABF2^+^* transformants (45±1%) was in the G1 phase of the cell cycle compared to the empty vector transformants (52±2%, Figure 2B). Thus, the cell cycle effects we observed in *3xABF2* ^+^ cells are *ABF2*-linked.

### Abf2p and the Retrograde Response

**Figure 3:**
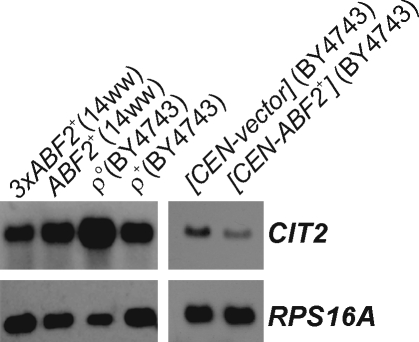
Moderate over-expression of *ABF2* does not trigger the RTG response. RNA blots of *CIT2* steady-state levels, from cells grown under glucose limitation in chemostats. The relevant genotype and strain background are shown above each lane. *RPS16A* levels indicate loading.

If mitochondria do not function properly a retrograde (RTG) response leads to elevated (∼10-fold) *CIT2* levels [21],[22]. As expected, in ρ° cells, which lack mtDNA, the *CIT2* RNA level was increased ∼5-fold over the level in ρ^+^ cells (Figure 3). In contrast, we found that *CIT2* mRNA levels are not elevated in cells over-expressing *ABF2*. Instead, *CIT2* levels are reduced by ∼2-fold (Figure 3). Thus, the mitochondria of cells over-expressing *ABF2* are not dysfunctional. Using a colony sectoring assay [23], we also found that the frequency of chromosome loss was 1.66% (n = 3,004) for *CEN-ABF2^+^* transformants, compared to 1.73% (n = 2,657) for the empty vector transformants. Therefore, over-expression of *ABF2* does not cause gross genome instability.

### Abf2p and the Timing of START

**Figure 4:**
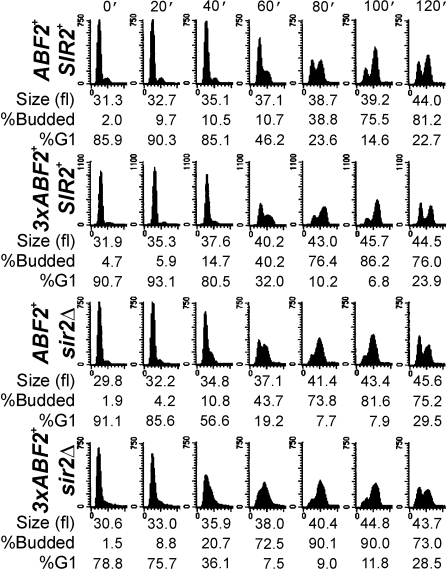
Over-expression of *ABF2*, or loss of *SIR2*, accelerates DNA replication. Synchronous cultures of the indicated strains were obtained by centrifugal elutriation and the starting populations of each strain were of the same size. At the indicated times the DNA content was evaluated by flow cytometry.

Because *3xABF2^+^* populations proliferate faster (Figure 1A) and have a reduced fraction of G1 cells (Figure 1B), we hypothesized that DNA replication may be accelerated in *3xABF2^+^* cells. We examined cell cycle progression of *ABF2^+^* and *3xABF2^+^* cells from synchronous cultures obtained by centrifugal elutriation. We used standard [24] undefined medium (YPD) for these experiments, with lower glucose concentration (0.5%). A higher fraction of *3xABF2^+^* cells entered S phase sooner than *ABF2^+^* cells (Figure 4, compare the top two rows). For example, at 60 min post-elutriation of *ABF2^+^* cells 10.7% were budded and 46.2% in G1, while of *3xABF2^+^* cells 40.2% were budded and 32% were in G1. In addition, the *3xABF2^+^* cells completed S phase sooner than *ABF2^+^* cells (see Figure 4 compare the top two rows): At 80 min post elutriation, note the size of the G2/M peak relative to the G1 peak. More *3xABF2^+^* cells have completed DNA replication than *ABF2^+^* cells. Finally, although in asynchronous populations the overall cell size of *3xABF2^+^* cells was not different from *ABF2^+^* cells (Figure 2A), the elutriated daughter *3xABF2^+^* G1 cells increased in size faster than their *ABF2^+^* counterparts (Figure 4, compare the top two rows): at 60 min *3xABF2^+^* cells are 40.2 fl, while *ABF2^+^* cells are 37.1 fl, consistent with a growth-promoting role of Abf2p.

To confirm these results, we repeated this analysis several times. In each case, *ABF2^+^* and *3xABF2^+^* cells were examined under identical conditions, using media from the same batch. We used two variables to compare the two strains across different experiments: the critical size for budding (at which 50% of the cells are budded); and the rate of cell size increase after elutriation. Interestingly, *3xABF2^+^* cells bud at a slightly larger size (41.2±1.1 fl, n = 5, *P* = 0.032, based on a 2-tailed Student's *t* test) than *ABF2^+^* cells (38.6±1.1 fl, n = 6) (Figure S1A and S1D). We then plotted in each case cell size as a function of time, to estimate the rate of cell size increase (fl/min) after elutriation (Figure S1B and S1C). While these values can vary from experiment to experiment, in every case *3xABF2^+^* cells increased in size significantly faster (0.14±0.02 fl/min, n = 5, *P* = 0.013, based on a 2-tailed Student's *t* test) than *ABF2^+^* cells (0.11±0.01 fl/min, n = 6) (Figure S1E). Thus, even though *3xABF2^+^* cells have a slightly larger critical size for budding than *ABF2^+^* cells, they reach that size significantly earlier than their *ABF2^+^* counterparts because they increase in size ∼28% faster. For example, for *ABF2^+^* newborn daughter cells of 20 fl, it will take on average 169 min until they reach their critical size, but it will take 150 min for *3xABF2^+^* daughters. Together with our chemostat experiments (Figures 1 and 2), our findings from these synchronous cultures (Figure 4 and Figure S1) with standard YPD media strongly support the notion that Abf2p plays an active growth-promoting role and accelerates initiation of DNA replication.

**Figure 5:**
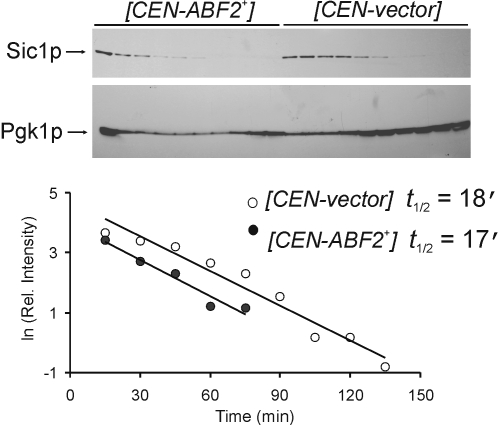
Sic1p stability and Abf2p. The levels of galactose-induced Sic1p-TAG were monitored by immunoblotting from cells carrying the indicated plasmids after *SIC1-TAG* expression was turned off. The signal from Pgk1p was used to estimate loading.

We also examined the levels of the cyclin-dependent kinase (Cdk) inhibitor Sic1p in cells over-expressing Abf2p. In late G1 rising levels of Cdk activity trigger the degradation of Sic1p and initiation of DNA replication [25]. In cells over-expressing Abf2p degradation of Sic1p was initiated sooner than in the control cells (Figure 5), consistent with a shortened G1 phase, but once triggered the rate of Sic1p degradation was unaffected. We obtained identical results in separate repeats of this experiment (Figure S2).

**Figure 6:**
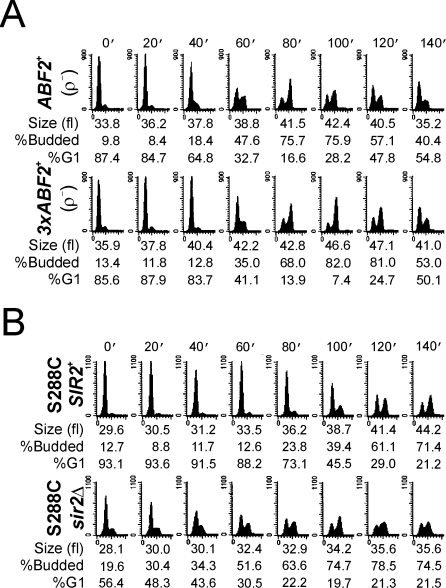
mtDNA is required for the accelerated DNA replication in *3xABF2^+^* cells. *A*, Cell cycle progression of elutriated cells was analyzed exactly as in Figure 4, except that the strains used were made ρ^−^ as described in the Methods. *B*, Loss of *SIR2* accelerates DNA replication in the S288C strain background. The elutriation experiment was done as in Figure 4, except that the strains used were in a different strain background, described previously [14].

We next generated the corresponding ρ^−^ strains to test whether over-expression of *ABF2^+^* requires mtDNA to promote DNA replication. These strains are respiratory incompetent (Figure S4A). DNA replication was not accelerated in *3xABF2^+^* (ρ^−^) cells (Figure 6A). Overall, in contrast to ρ^+^ cells (see Figure 4 and Figure S1) the critical budding size (Figure S4B), and the rate of cell size increase after elutriation (Figure S4C), were not significantly different between *ABF2^+^* (ρ^−^) and *3xABF2^+^* (ρ^−^) cells: *P* = 0.43, and *P* = 0.54, respectively (based on 2-tailed Student's *t* tests). In conclusion, our findings suggest that altered mitochondrial functions in *3xABF2^+^* cells impact on some factor(s) that affect DNA replication.

### Functional Interactions between Abf2p and Sir2p

**Figure 7:**
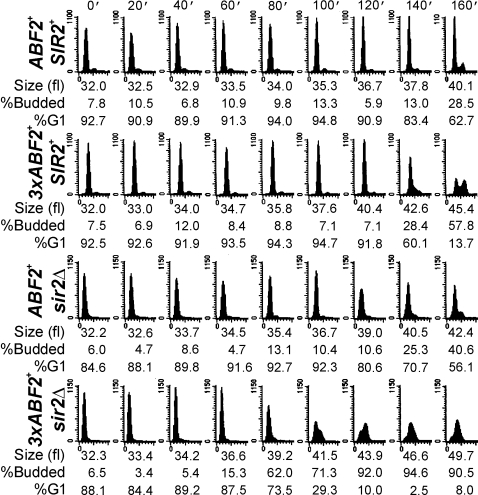
Loss of *SIR2* in cells over-expressing *ABF2* dramatically accelerates DNA replication when NADH is depleted. Cell cycle progression was monitored after elutriation as in Figure 4, except that 10 mM acetaldehyde was added to the starting samples.

A protein linked to both metabolism and DNA replication is the Sir2p sirtuin [11], which negatively impacts DNA replication [16]–[18]. Consequently, we evaluated cell cycle progression of cells lacking Sir2p alone, or in combination with Abf2p over-expression (Figure 4). Comparison of *ABF2^+^*, *SIR2^+^* (Figure 4, top row) to *ABF2^+^*, *sir2Δ* (Figure 4, third row) cells at 60 min shows that cells lacking *SIR2* initiated and completed S phase significantly sooner than wild type cells. Initiation of DNA replication was further accelerated in *3xABF2^+^*, *sir2Δ* cells (Figure 4, bottom row). We repeated this analysis several times, as we described earlier (Figure S1). Interestingly, *3xABF2^+^*, *sir2Δ* cells bud at a smaller size (36.1±0.5 fl, n = 5, *P* = 0.0009, based on a 2-tailed Student's *t* test) than *ABF2^+^*, *SIR2^+^* cells (38.6±1.1 fl, n = 6) (Figure S1D). This explains the apparent additive acceleration of START we observed in *3xABF2^+^*, *sir2Δ* cells (Figure 4, compare at 40 min the *3xABF2^+^*, *sir2Δ* strain to other strains, and see also Figure 7, below). Taking into account the critical budding size and the rate of cell size increase for each strain, for *3xABF2^+^*, *sir2Δ* newborn daughter cells of 20 fl, it will take on average 128 min to start budding, compared to 169 min for *ABF2^+^*, *SIR2^+^* daughters. Finally, loss of Sir2p does not increase the amount of mtDNA in the cell (Figure S3).

We next generated the corresponding (ρ^−^) strains lacking Sir2p (Figure S4A). These strains were examined after elutriation (Figure S4B and S4C), as we described above. Strains lacking Sir2p and mtDNA did not have a significant different rate of cell size increase after elutriation compared to the other (ρ^−^) strains (Figure S4C). Finally, to ensure that the effects of Sir2p on cell cycle progression were not strain-specific, we also examined cell cycle progression of *sir2Δ* cells in a different strain background (an S288C derivative) [14]. S phase entry was greatly accelerated in *sir2Δ* cells in that background, and cells spent very little time in G1 (Figure 6B). For example, *SIR2^+^* cells initiate DNA replication at 80–100 min after elutriation, while *sir2Δ* cells do so at ∼40 min.

We next tested if the acceleration of DNA replication in *3xABF2^+^* or *sir2Δ* cells depends on NADH. Yeast cells can display robust NAD(P)H oscillations, which are thought to gate metabolism with DNA replication, since DNA synthesis takes place when NAD(P)H levels are high [26]–[28]. To deplete cellular NADH we added 10 mM acetaldehyde to the elutriated early-G1 cells [29]. The G1 phase was greatly expanded (Figure 7), compared to the untreated cells shown in Figure 4, consistent with a requirement for NADH for initiation of DNA replication. G1 phase expansion was also evident in *3xABF2^+^*, *SIR2^+^* or *ABF2^+^*, *sir2Δ* cells, indicating that these cells still require NADH to progress through G1 into S phase. Nonetheless, *3xABF2^+^*, *SIR2^+^* or *ABF2^+^*, *sir2Δ* cells entered S phase sooner (∼20 min) than wild type *ABF2^+^*, *SIR2^+^* cells (Figure 7, compare the top three rows at 140 min post-elutriation), consistent with our previous results shown in Figure 4. Remarkably, 3x*ABF2^+^*, *sir2Δ* cells entered and completed S phase with highly accelerated kinetics: they finished DNA replication before wild type cells even started (Figure 7, compare the bottom row with the top row). These results are consistent with strong additive functional interactions between Abf2p and Sir2p, with Sir2p acting antagonistically to Abf2p's effects on DNA replication. How Abf2p over-expression impacts the metabolic status of the cell is unclear, but it may involve NAD/NADH metabolism because the functional interactions between Abf2p and Sir2p are quite prominent in the presence of acetaldehyde.

**Figure 8:**
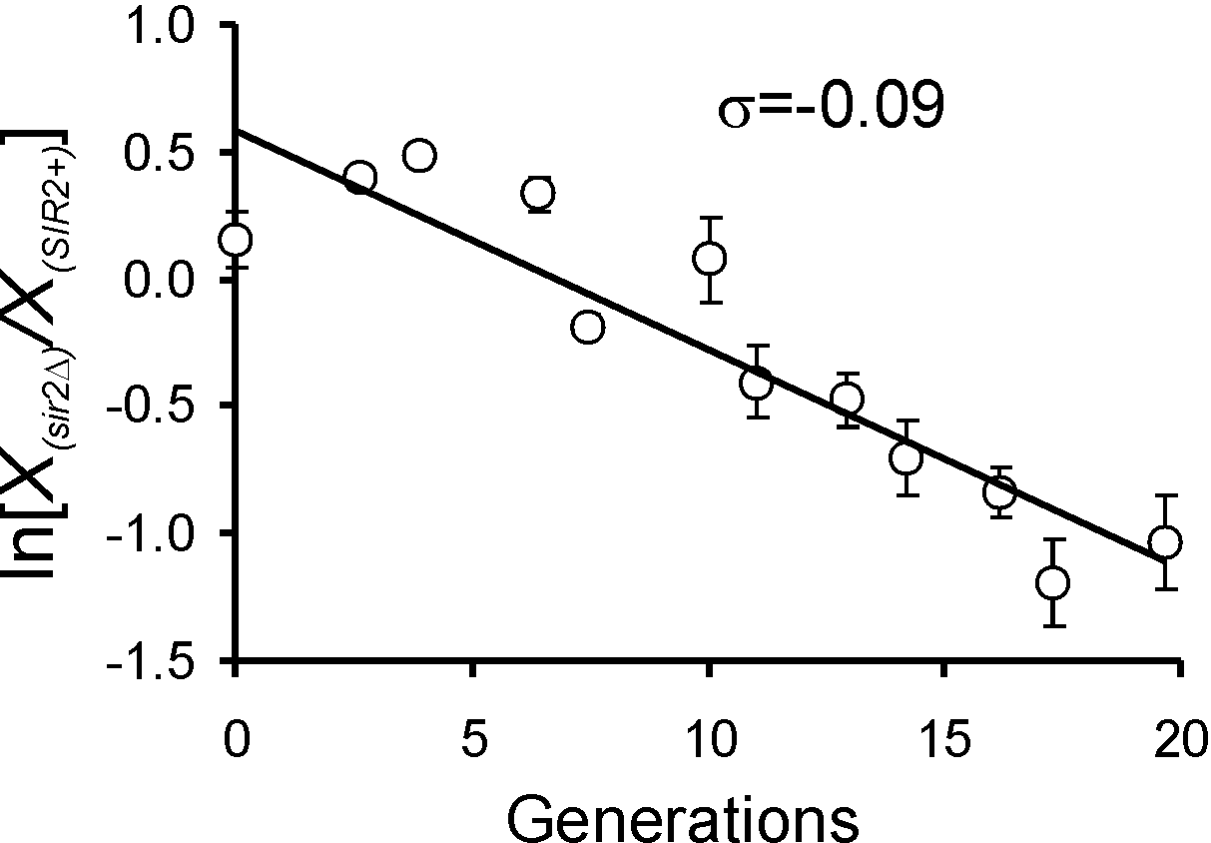
Loss of Sir2p does not accelerate overall cell proliferation. Chemostat competition experiments between *SIR2^+^* and *sir2Δ* cells (in the 14ww strain background) were done during glucose limitation at dilution rate *D* = 0.1 h^−1^, as described in Figure 1.

We next asked if Sir2p negatively affects cellular metabolism to delay DNA replication. We found that *sir2Δ* cells did not proliferate faster than *SIR2^+^* cells under glucose limitation in chemostats (Figure 8). Thus, loss of Sir2p does not up-regulate metabolic functions necessary to achieve the proliferation advantage evident in *3xABF2^+^* cells under the same conditions (Figure 1A).

### Abf2p and Physical Changes at Origins of DNA Replication

**Figure 9:**
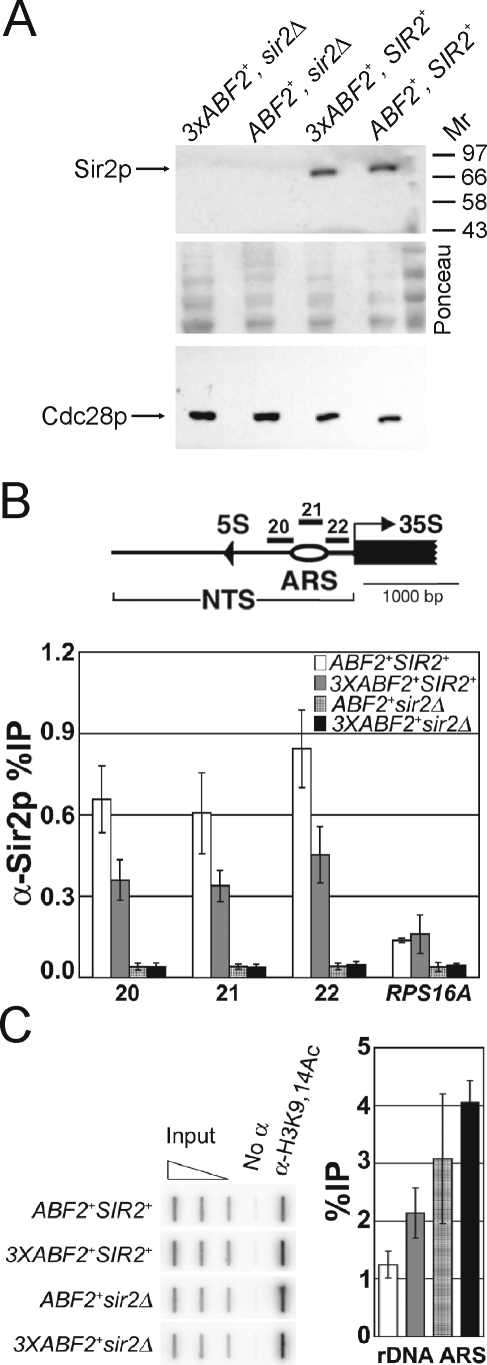
Cells over-expressing *ABF2* have less Sir2p and higher levels of K9, K14 acetylated histone H3 at the rDNA ARS elements. *A*, Immunoblot showing that the level of Sir2p is not altered in whole cell extracts from cells over-expressing *ABF2*. The same blot was stained with Ponceau, to indicate loading. Loading was also evaluated from the same samples, with an anti-Cdc28p antibody. *B*, ChIP experiments analyzed by real-time PCR show that the level of Sir2p (%IP) at the rDNA ARS elements is reduced in *3XABF2^+^* cells. Part of one rDNA repeat is shown above indicating the location of the rDNA ARS elements, the primers used (primer pairs 20, 21, 22); the nontranscribed spacer (NTS); and the 35S and 5S rRNA genes. The values shown in the bar graph are the average %IPs (±s.d.) of three independent experiments. *C*, ChIP experiments analyzed by slot blot show that the level of K9, K14 acetylated histone H3 is increased at the rDNA ARS elements in *3XABF2^+^* cells. In the graph, the average %IPs (+/− range) are shown for two independent experiments (Pearson coefficient = 0.95). The open triangle above the slot blot represents serial dilutions of input DNA to ensure linearity with respect to hybridization of the probe to the amount of DNA applied to the membrane. Other labels as in Figure 9B.

The overall levels of Sir2p are not altered in *3xABF2^+^* cells (Figure 9A). In addition to its roles in silencing, Sir2p negatively affects the activity of origins of DNA replication throughout the genome [16],[17]. Consequently, we next tested if the level of Sir2p at origins of DNA replication is altered in *3xABF2^+^* cells.

We examined the ARS elements in the rDNA tandem repeats, because the association of Sir2p with the rDNA [30] and the negative role of Sir2p in regulating these origins [16],[17] are well-characterized. Chromatin immunoprecipitation (ChIP) was performed with *ABF2^+^* or *3xABF2^+^* cells using antisera against Sir2p. *ABF2^+^*, *sir2Δ* cells and *3xABF2^+^*, *sir2Δ* cells were examined to provide a measurement of background. Immunoprecipitated DNA was analyzed by real-time PCR using primers that span the ARS elements in the rDNA. We found that the level of Sir2p at the rDNA ARS elements was reduced about two-fold in *3xABF2^+^* cells, compared to the level in *ABF2^+^* cells (Figure 9B). The level of Sir2p at *RPS16A*, a locus that does not contain an ARS element, was not altered by over-expression of Abf2p. Consistent with the reduced level of Sir2p at the rDNA ARS elements, we also found that the level of K9, K14-acetylated histone H3 at the rDNA ARS elements was increased in cells over-expressing Abf2p (Figure 9C). In yeast and animals such chromatin modifications activate DNA replication [18],[19],[31]. In addition to the rDNA ARS we also examined ARS315, which is a very active origin and fires in 90% of the cell cycles [32]. Consistent with the high activity of ARS315, the level of K9, K14-acetylated histone H3 was also very high at that origin (data not shown). Since loss of Sir2p suppresses replication defects of ARS315 in *cdc6-4* cells [17], we then examined if Sir2p is present at ARS315. We did not detect Sir2p at ARS315 in *ABF2^+^* or *3xABF2^+^* cells (Figure S5), perhaps consistent with the already high activity of this origin. Thus, the previously observed [17] effects of Sir2p on MCM proteins binding at ARS315 maybe indirect.

**Figure 10:**
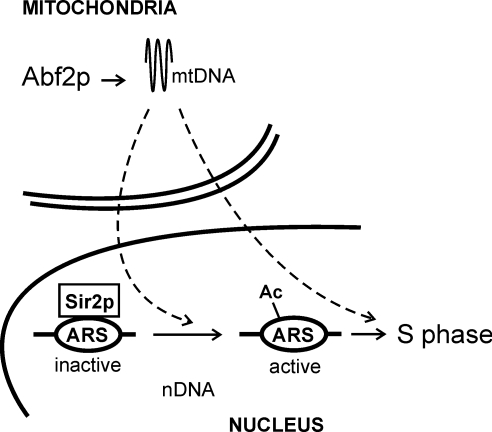
A schematic of the model suggested by our data. An increase of mtDNA by moderate over-expression of Abf2p promotes mitochondrial functions, which in turn accelerate cell proliferation and DNA replication. The NAD^+^-dependent de-acetylase Sir2p antagonizes initiation of DNA replication. Cells over-expressing Abf2p have decreased levels of Sir2p at origins of DNA replication (ARS), and higher levels of acetylated K9, K14 histone H3 residues (Ac). Additional mechanisms likely contribute to the overall positive effects on cell proliferation and DNA replication in cells with higher mtDNA levels.

To answer if binding of Sir2p at the rDNA origins depends on the presence of mtDNA, we then examined the corresponding ρ^−^ strains (Figure S6). Notably, in both *ABF2^+^* or *3xABF2^+^* ρ^−^ cells Sir2p levels are increased at the rDNA ARS by ∼2-fold (Figure S6). Thus, the level of Sir2p bound at the rDNA ARS elements is inversely related to the amount of mtDNA in the cell. It appears that some mitochondrial function that depends on mtDNA limits the association of Sir2p with origins of replication. Together, these results identify physical changes associated with active origins of DNA replication in the nucleus, resulting from an increase in the amount of the mitochondrial genome (Figure 10).

In conclusion, the control of DNA replication by an increase in mtDNA we describe here suggests that the mitochondrion does not simply provide the energy at the service of its larger cellular host, but it may actively dictate when cells initiate their division. Furthermore, metabolic control of chromatin modifications may provide critical links between metabolism and cell division.

## Materials and Methods

### Strains and Plasmids

The haploid *3xABF2^+^* strain and its wild-type counterpart (14ww) have been described elsewhere [9]. Replacement of *SIR2* with a *sir2Δ*::KanMX6 cassette was done by standard methodology [33]. BY4743 is a standard diploid strain [34]. The strains used in Figure 3 were made ρ° after three passages of single colonies on plates containing 25 µg/ml ethidium bromide. A single colony from the final ethidium bromide passage was then plated on glucose and glycerol-containing plates to ensure the isolated colony was respiratory deficient and no growth occurred on the glycerol plates. Further PCR analysis failed to detect the presence of the *COX2* gene, which is mtDNA-encoded, in the ρ° strains, but it detected the *CIT2* gene, which is nDNA-encoded. We similarly generated the ρ^−^ strains used in Figure 6A, Figure S4, and Figure S6.

For the Sic1p stability experiment described in Figure 5 and Figure S2, the strains used were diploids, obtained from a cross of YSC3869-9515050 ([P*_GAL_-SIC1*-*TAG*::*URA3* [35]], BY4741 [34] otherwise; purchased from Open Biosystems), with strain BY4742 [34] carrying (*CEN-ABF2^+^*) [9],[20], or the empty vector (*CEN*-*vector*).

### Yeast Cultivation

For batch cultures we followed established yeast protocols [24]. Conditions for chemostat cultures have been described previously [36],[37].

### Elutriation

We followed previously described protocols to obtain cell cycle parameters [38], except that the cells were cultured and collected in YPD (0.5% glucose) medium. The percentage of cells in G1 from the flow cytometry panels was calculated from the DNA histograms using the ModFit® software (Verity Software House, ME). Cell cycle progression was also monitored microscopically, by the percentage of budded cells. Cell size (fl) was measured using a channelyzer.

From each elutriation experiment, we plotted the percent of budded cells as a function of cell size. To estimate the critical budding size, when ∼50% cells are budded, we used data points from the linear portion of each graph, which were fit to a straight line using the regression function in Microsoft Excel. From the resulting equation [(%budded) = a(Cell size)-b; where a is the slope and b the y-intercept of the line] we calculated the critical budding size for each experiment. The average of all experiments for each strain was then calculated, along with the associated standard deviation.

From the same elutriations, to calculate the rate of cell size increase, we plotted the cell size as a function of time. The data were also fit to a straight line using the regression function in Microsoft Excel. From the slope of the line we obtained the rate of cell size increase. The average of all experiments for each strain was then calculated, along with the associated standard deviation.

### RNA Methods

Standard protocols [24] were used for RNA extraction and electrophoresis. The *CIT2* and *RPS16A* probes (see Figure 3) were generated by PCR, and labeled using non-radioactive reagents from the North2South® Biotin Random Prime Labeling Kit (Pierce), according to their instructions. Probe hybridization and detection were performed according to the North2South® Chemiluminescent Hybridization and Detection Kit (Pierce).

### mtDNA Abundance

We used PCR to estimate the relative amount of mtDNA (see Figure S3). The exponential range of amplification for the *COX2* (mtDNA-encoded) and *GID8* (nDNA-encoded) PCR products were determined by performing separate reactions for each of the two amplified products and removing them after 25, 30, or 35 cycles. The PCR products were run on a 2% agarose gel and the ethidium-stained signal intensities for the 30-cycle products (which were in the linear range of amplification) were quantified with Adobe Photoshop®. The ratio between the *COX2* and the *GID8* product was determined to normalize for differences in initial DNA concentration and reaction efficiencies. The ratio for each strain was then compared to *ABF2^+^* cells to determine the relative amounts of mtDNA.

### Sic1p Stability Assays

Cultures were diluted 10-fold from an overnight culture in selective liquid synthetic complete medium containing raffinose as carbon source, to a total volume of 10 ml. Cells were grown at 30°C, with shaking for 30–48 h to synchronize the cells in G1 by starvation. Galactose was then added to 2% (w/v) final concentration for 2 to 3 hours to induce expression of P*_GAL_-SIC1*-*TAG*. After induction, the cells were pelleted, washed in water, and re-suspended in 20 ml fresh medium containing 2% (w/v) glucose, to stop expression of P*_GAL_-SIC1*-*TAG*. The cells were cultured at 30°C and 1 ml was taken every 15 min, to monitor Sic1p-TAG levels. Proteins were extracted using TCA precipitation and resolved by SDS-PAGE. For immunoblotting, we used the PAP reagent (Sigma, used according to their instructions) to detect the Protein A epitope present in Sic1p-TAG. The same blot was also probed with an anti-Pgk1p antibody (from Molecular Probes, and used according to their instructions). Pgk1p is not cell cycle regulated, and it is widely used as a loading control in cell cycle experiments, including landmark studies that accurately quantified the levels of cell cycle proteins [39]. The intensity of the bands was quantified using Adobe Photoshop, normalizing the levels of Sic1p-TAG to the loading control. Using Microsoft Excel, values were fit to a linear transformation of the exponential decay equation (lnX*_t_* = −*kt*+lnX*_t0_*, where X denotes the amount of Sic1p-TAG, *k* the degradation rate constant, and *t* time) to obtain *k* from the slope of the line. The half-life of Sic1p-TAG was then determined from *t*_1/2_ = ln2/*k*.

### Chromatin Immunoprecipitation

For these analyses, cells were cultured in YPD containing 0.5% glucose. The primers and protocols for ChIP experiments and analysis have been described previously [13],[40]. ChIPs were performed with anti-Sir2p antisera (Santa Cruz Biotechnology, Cat#: sc-6666) or anti H3K9, K14Ac (Millipore, Cat#: 06-599). Percent immunoprecipitations (%IP) were determined by dividing IP signal by input signal. ChIP experiments were analyzed either by quantitative PCR (Figure 9B and Figure S5), or by slot blot (Figure 9C and Figure S6). For anti-Sir2p ChIPs, chromatin from *sir2Δ* cells was analyzed to assess background signal. For anti H3K9, K14Ac ChIPs, “no antibody” controls were included to assess background signal. For slot blot analysis, samples were blotted to a membrane that was hybridized to a ^32^P-labeled probe spanning the rDNA ARS region. Signals were quantified on a Storm 860 phosphorimager (Molecular Dynamics) using Imagequant software. Ethidium-stained gels were quantified using Quantity One software (BioRad).

### Other

For Sir2p immunoblotting (Figure 9A) we used anti-Sir2p antisera (Cat#: sc-6666) from Santa Cruz Biotechnology, and a secondary antibody from Pierce, at the recommended dilutions. The anti-Cdc28p antibody used to estimate loading was also from Santa Cruz Biotechnology (Cat#: sc-6708), and used according to their instructions. The immunoblots were processed with reagents from Pierce.

## Supporting Information

Figure S1 Summary of data from elutriation experiments with rho^+^ strains. A, The raw data points showing the percent of budded cells as a function of cell size, from separate independent elutriation experiments with ABF2^+^ and 3XABF2^+^ strains. The data points shown were from the linear portion of each experiment, when the percentage of budded cells began to increase, and they were used to determine the critical budding size as described in Materials and Methods. B,C, The rate of cell size increase for each elutriation experiment of the indicated strains (ABF2^+^ and 3XABF2^+^) is shown. From these graphs we calculated the rate of size increase as described in Materials and Methods. D, The critical size for budding of the indicated strains is shown. Where marked with an asterisk (*), the difference is statistically significant, based on 2-tailed Student's t tests. E, The rate of cell size increase of the indicated strains is shown. Where marked with an asterisk (*), the difference is statistically significant, based on 2-tailed Student's t tests.
(0.06 MB TIF)

Figure S2 Sic1p stability and Abf2p. A separate experiment, similar to the one described in Fig. 5, is shown, except that loading was estimated from the Ponceau-stained blot.
(0.23 MB TIF)

Figure S3 mtDNA abundance is not increased in cells lacking Sir2p. To estimate the mtDNA abundance of the indicated strains we used PCR (Top), as described in Materials and Methods. The ratio between the COX2 and the GID8 product was determined to normalize for differences in initial DNA concentration and reaction efficiencies. The ratio for each strain relative to ABF2^+^ cells is shown (Bottom). Graph represents average data from two independent experiments (+/− range). As a control, we also performed this analysis on 3XABF2^+^ cells, which are known to have higher mtDNA levels.
(0.11 MB TIF)

Figure S4 Cell cycle progression of rho^−^ strains. A, The strains used were respiratory-incompetent and they could not proliferate on plates with glycerol as a carbon source. The critical size for budding (B), and the rate of cell size increase (C), of the indicated strains was determined as in Figure S1.
(0.40 MB TIF)

Figure S5 ChIP experiments from the indicated strains analyzed by PCR do not detect Sir2p bound to ARS315 (left panel). As a control, we also performed this analysis on rDNA ARS using primer pair 21 and detected Sir2p association with the rDNA ARS (right panel). PCR products from input and IP samples were subjected to agarose gel electrophoresis and analyzed by ethidium bromide staining. The open triangles represent serial dilution of template DNA in the PCR reaction. Other labels as in Fig. 9B.
(0.23 MB TIF)

Figure S6 Sir2p ChIP to rDNA ARS in rho^+^ and rho^−^ cells. ChIP experiments from the indicated strains analyzed by slot blot to detect Sir2p bound to rDNA ARS. Note that applying slot blot methodology to the rDNA ARS reproduced the reduced Sir2p levels bound to the rDNA ARS in 3XABF2^+^ cells that we observed with the real-time PCR analysis shown in Fig. 9B. Graph represents average data from two independent experiments (+/− range). Other labels as in Fig. 9.
(0.26 MB TIF)
