# Correction: An Increase in Mitochondrial DNA Promotes Nuclear DNA Replication in Yeast

**DOI:** 10.1371/annotation/89355dbd-390e-463f-b804-c6cf1296402e

**Published:** 2008-05-13

**Authors:** Heidi M. Blank, Chonghua Li, John E. Mueller, Lydia M. Bogomolnaya, Mary Bryk, Michael Polymenis

Mary Bryk should also be listed as a corresponding author and should be contacted regarding chromatin aspects. Her e-mail address is bryk@tamu.edu.

