# Supplementary figures and images for: An Increase in Mitochondrial DNA Promotes Nuclear DNA Replication in Yeast
Source: PLoS Genet. 2008 Apr 25;4(4):e1000047. doi: 10.1371/journal.pgen.1000047 (PMC2289842; doi:10.1371/journal.pgen.1000047)

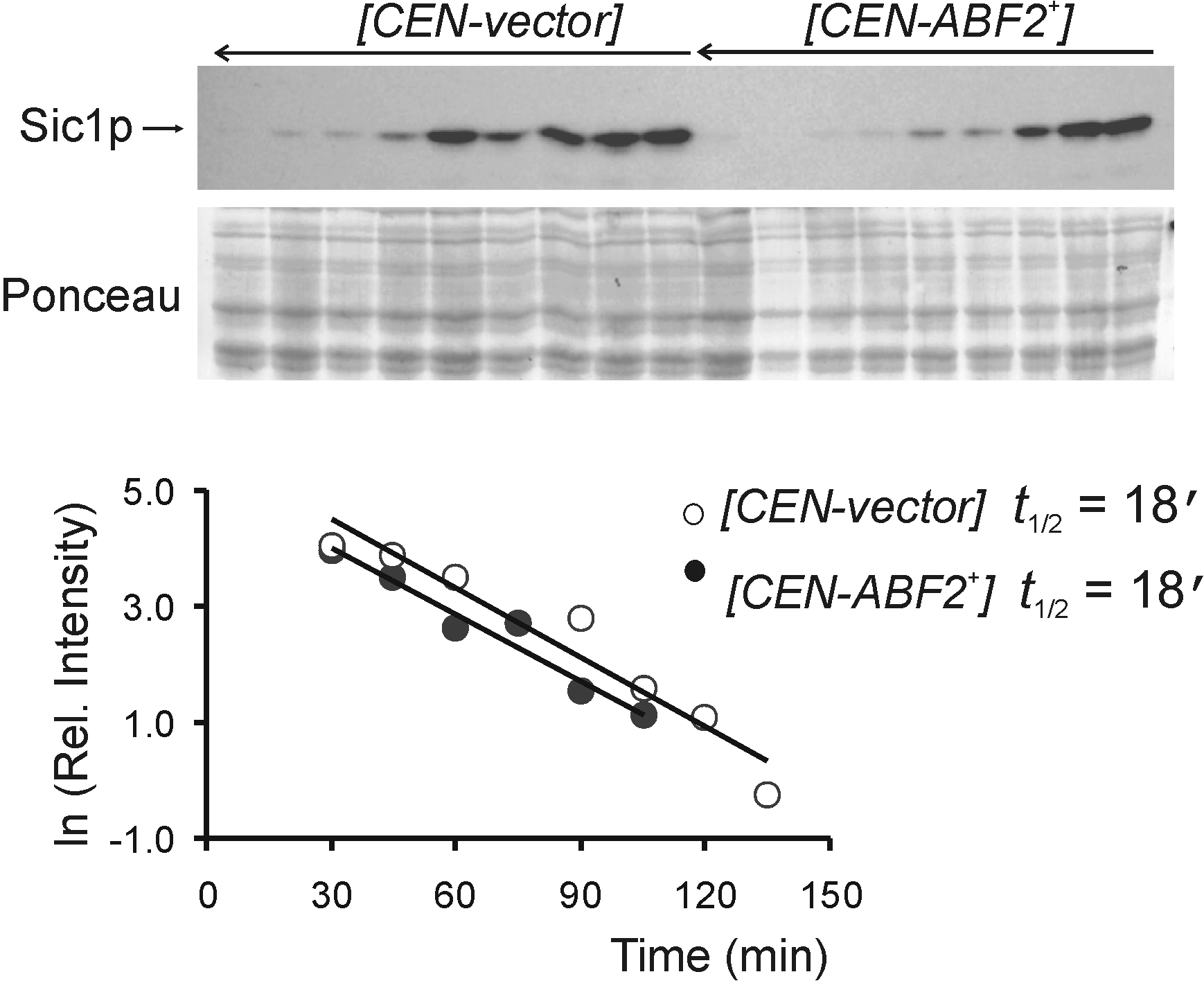

Supplement: Figure S2 — Sic1p stability and Abf2p. A separate experiment, similar to the one described in Fig. 5, is shown, except that loading was estimated from the Ponceau-stained blot. (0.23 MB TIF) [file pgen.1000047.s002.tif]

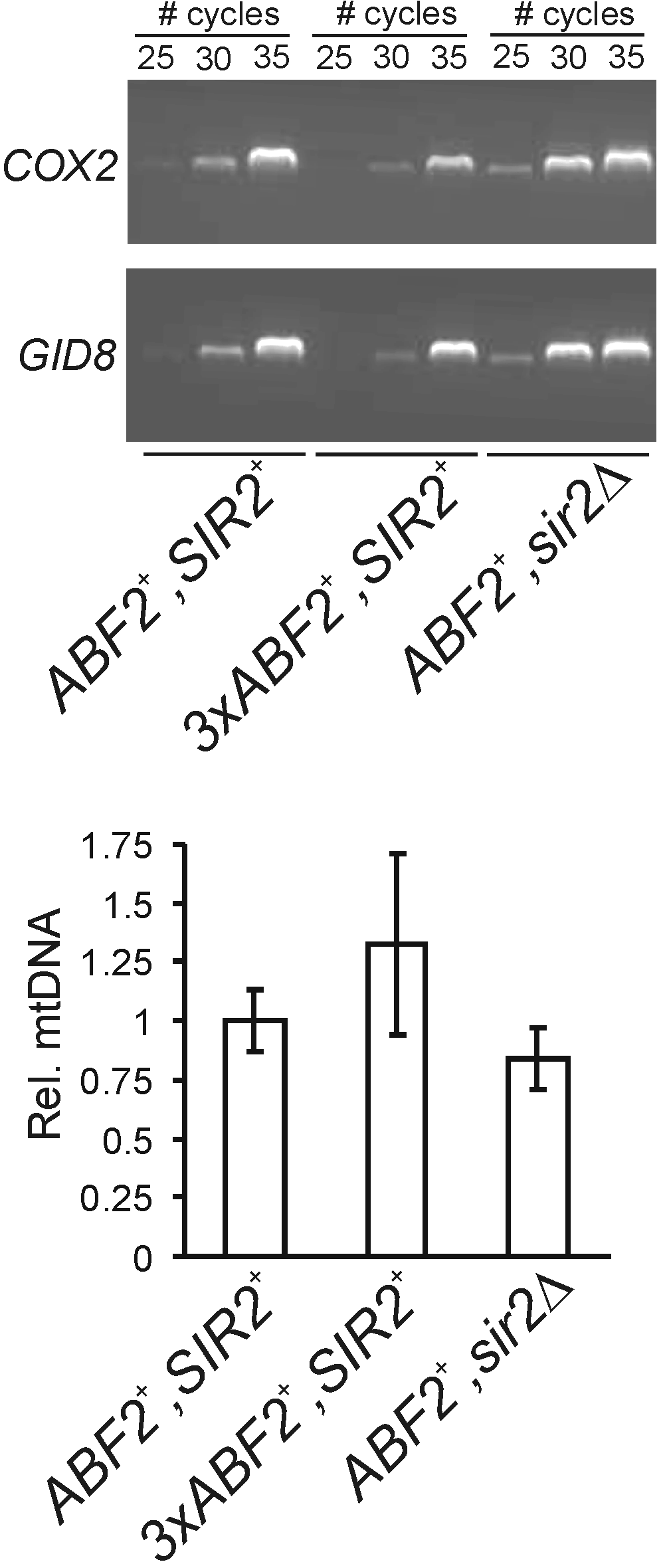

Supplement: Figure S3 — mtDNA abundance is not increased in cells lacking Sir2p. To estimate the mtDNA abundance of the indicated strains we used PCR (Top), as described in Materials and Methods. The ratio between the COX2 and the GID8 product was determined to normalize for differences in initial DNA concentration and reaction efficiencies. The ratio for each strain relative to ABF2+ cells is shown (Bottom). Graph represents average data from two independent experiments (+/− range). As a control, we also performed this analysis on 3XABF2+ cells, which are known to have higher mtDNA levels. (0.11 MB TIF) [file pgen.1000047.s003.tif]

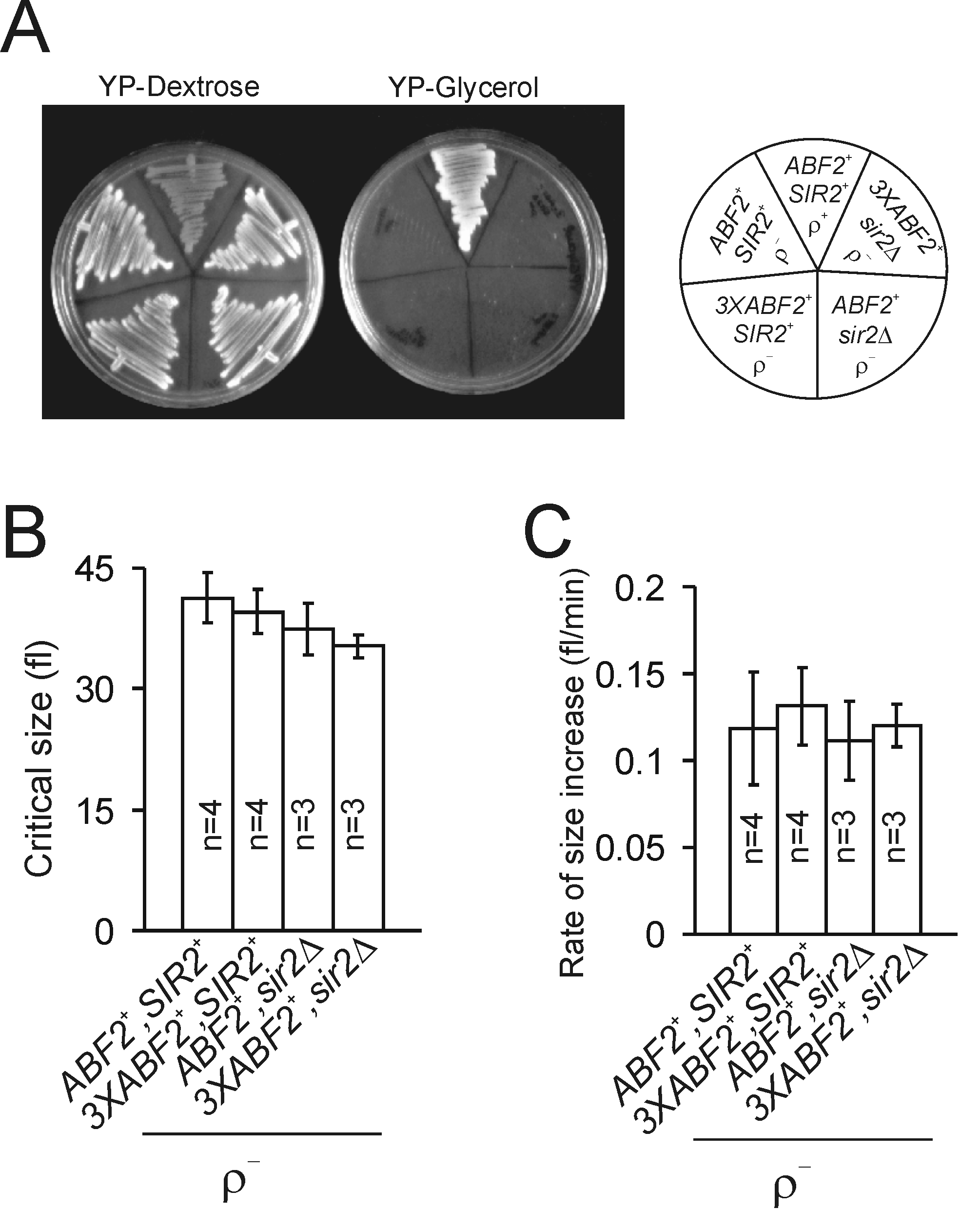

Supplement: Figure S4 — Cell cycle progression of rho− strains. A, The strains used were respiratory-incompetent and they could not proliferate on plates with glycerol as a carbon source. The critical size for budding (B), and the rate of cell size increase (C), of the indicated strains was determined as in Figure S1. (0.40 MB TIF) [file pgen.1000047.s004.tif]

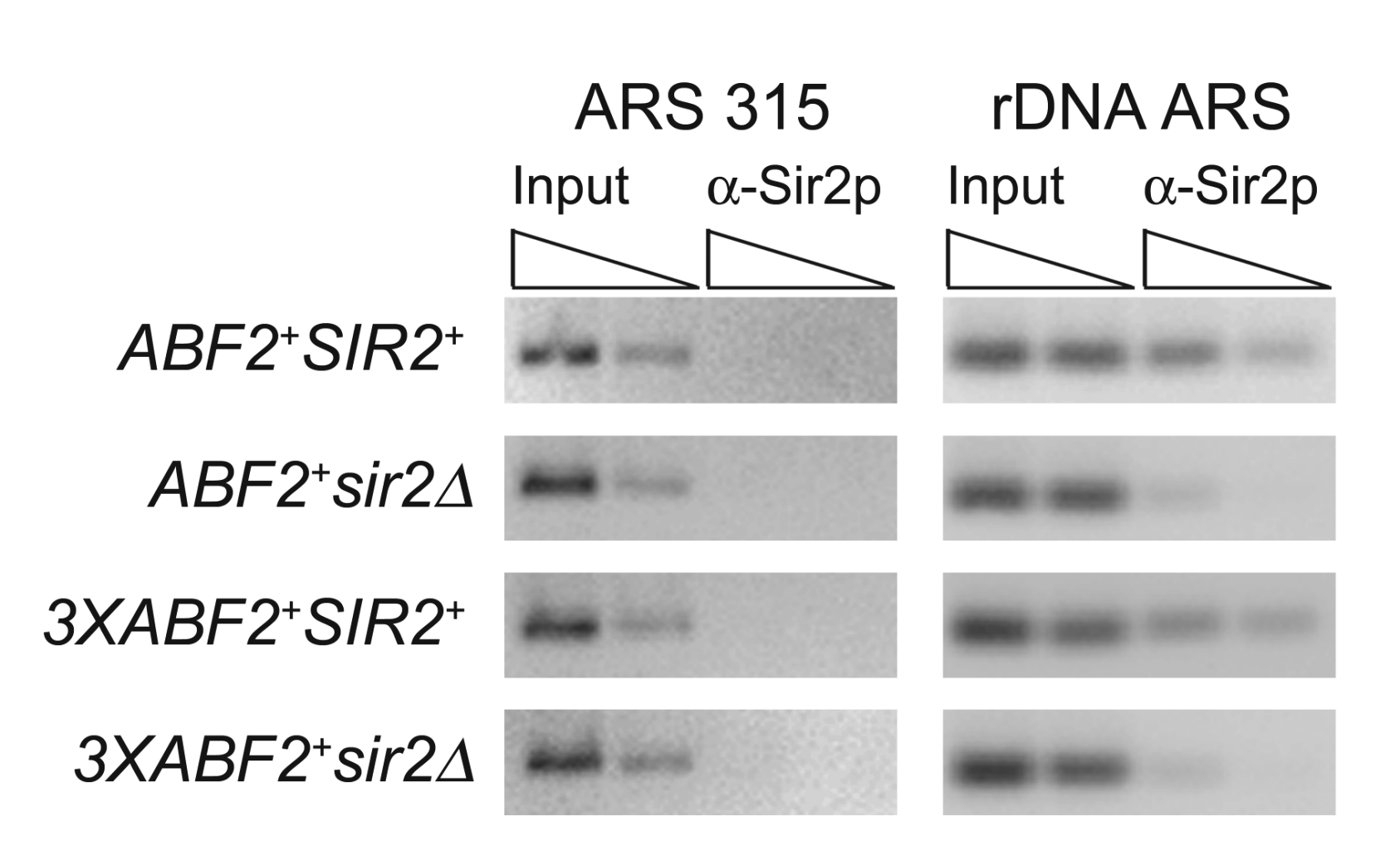

Supplement: Figure S5 — ChIP experiments from the indicated strains analyzed by PCR do not detect Sir2p bound to ARS315 (left panel). As a control, we also performed this analysis on rDNA ARS using primer pair 21 and detected Sir2p association with the rDNA ARS (right panel). PCR products from input and IP samples were subjected to agarose gel electrophoresis and analyzed by ethidium bromide staining. The open triangles represent serial dilution of template DNA in the PCR reaction. Other labels as in Fig. 9B. (0.23 MB TIF) [file pgen.1000047.s005.tif]

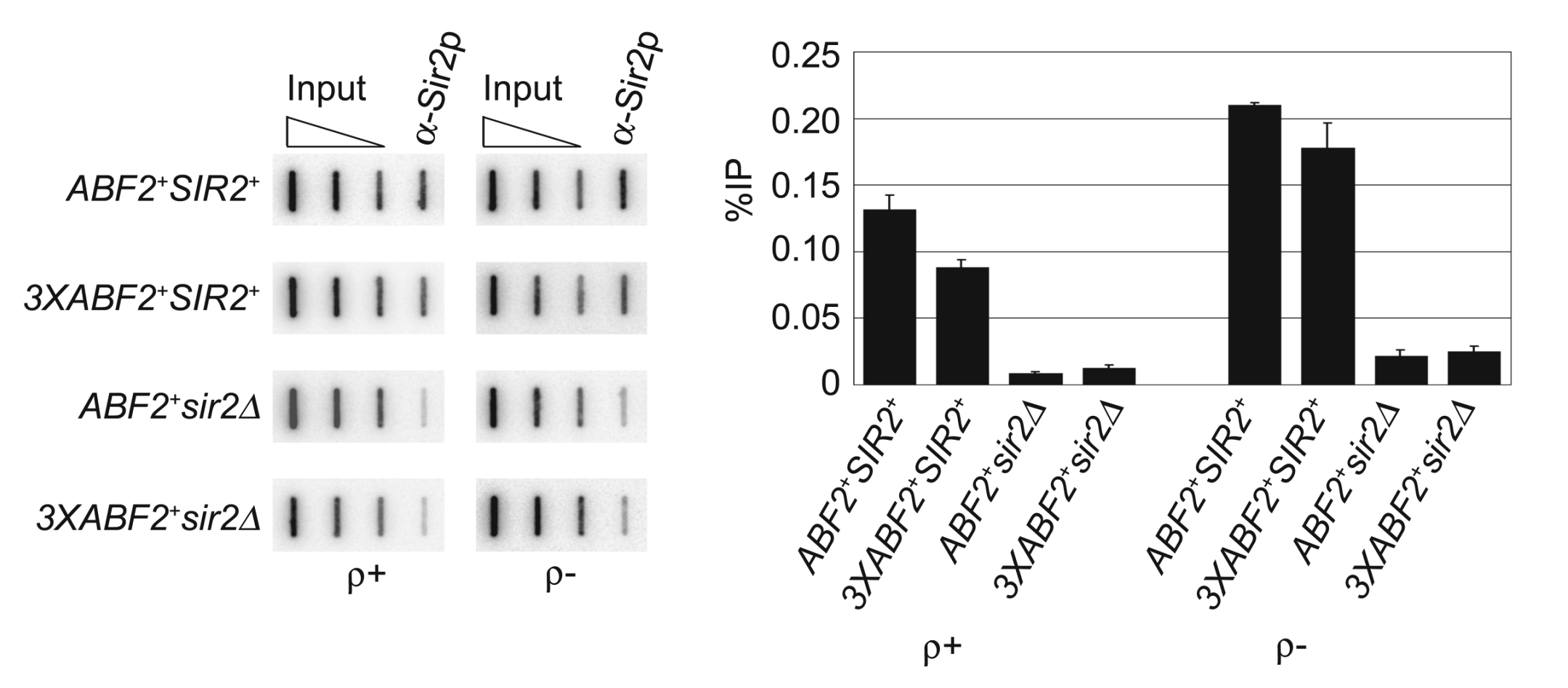

Supplement: Figure S6 — Sir2p ChIP to rDNA ARS in rho+ and rho− cells. ChIP experiments from the indicated strains analyzed by slot blot to detect Sir2p bound to rDNA ARS. Note that applying slot blot methodology to the rDNA ARS reproduced the reduced Sir2p levels bound to the rDNA ARS in 3XABF2+ cells that we observed with the real-time PCR analysis shown in Fig. 9B. Graph represents average data from two independent experiments (+/− range). Other labels as in Fig. 9. (0.26 MB TIF) [file pgen.1000047.s006.tif]
